# The association between XRCC3 rs1799794 polymorphism and cancer risk: a meta-analysis of 34 case–control studies

**DOI:** 10.1186/s12920-021-00965-4

**Published:** 2021-04-30

**Authors:** Weiqing Liu, Shumin Ma, Lei Liang, Zhiyong Kou, Hongbin Zhang, Jun Yang

**Affiliations:** 1 Department of Internal Medicine-Oncology, the First Affiliated Hospital of Kunming Medical University, Yunnan Province, Kunming, 650032 People’s Republic of China; 2 Department of Oncology, the First Affiliated Hospital of Kunming Medical University, Yunnan Province, No. 295 Xichang Road, Kunming, 650032 People’s Republic of China

**Keywords:** Rs1799794, Polymorphism, Cancer, Risk, Meta-analysis

## Abstract

**Background:**

Studies on the XRCC3 rs1799794 polymorphism show that this polymorphism is involved in a variety of cancers, but its specific relationships or effects are not consistent. The purpose of this meta-analysis was to investigate the association between rs1799794 polymorphism and susceptibility to cancer.

**Methods:**

PubMed, Embase, the Cochrane Library, Web of Science, and Scopus were searched for eligible studies through June 11, 2019. All analyses were performed with Stata 14.0. Subgroup analyses were performed by cancer type, ethnicity, source of control, and detection method. A total of 37 studies with 23,537 cases and 30,649 controls were included in this meta-analysis.

**Results:**

XRCC3 rs1799794 increased cancer risk in the dominant model and heterozygous model (GG + AG vs. AA: odds ratio [OR] = 1.04, 95% confidence interval [CI] = 1.00–1.08, P = 0.051; AG vs. AA: OR = 1.05, 95% CI = 1.00–1.01, P = 0.015). The existence of rs1799794 increased the risk of breast cancer and thyroid cancer, but reduced the risk of ovarian cancer. In addition, rs1799794 increased the risk of cancer in the Caucasian population.

**Conclusion:**

This meta-analysis confirms that XRCC3 rs1799794 is related to cancer risk, especially increased risk for breast cancer and thyroid cancer and reduced risk for ovarian cancer. However, well-designed large-scale studies are required to further evaluate the results.

## Background

Cancer is the leading cause of death worldwide, and the number of patients with cancer is increasing [1]. The occurrence of cancer is related to many factors, including environmental, lifestyle, genetic and other factors. Among them, gene mutation is a kind of genetic factor, which has a great influence on cancer risk [2]. The mutation in BRCA1 and BRCA2 is related to the increase risk of breast cancer [3]. XPF rs2276466 polymorphism is related to neurogenic cancer [4].

X-ray repair cross-complementing group 3 (XRCC3), functions in the homologous recombination (HR) repair of DNA crosslinks [5] and double-strand breaks [6]. Based on the function of XRCC3, XRCC3 gene mutations are related to the occurrence and development of many diseases. For example, XRCC3 241Thr/Met genotype promotes left ventricular hypertrophy by inhibiting DNA damage repair [7]. Mutations in the XRCC3 gene affect mitochondrial DNA integrity [8]. XRCC3 rs861539 polymorphism is associated with poor prognosis of breast cancer patients [9]. The mutation sites that have been studied more about the relationship between XRCC3 gene and cancer are rs861539, rs1799794 and rs1799796 [10]. However, results remain fairly conflicting rather than conclusive. A number of meta-analyses have investigated the relationship between rs861539 and susceptibility to various cancers [11–33]. However, there have been few meta-studies on rs1799794 and susceptibility to cancer [28, 30, 31, 33, 34]. Therefore, we conducted this meta-analysis to analyze the relationship between rs1799794 and susceptibility to cancer on the basis of more data.

## Methods

### Search strategies

We comprehensively searched five databases (PubMed, Embase, the Cochrane Library, Web of Science, and Scopus) for research published as of June 11, 2019, using relevant MeSH terms and entry terms. The keywords of XRCC3 included X-ray repair cross complementing 3, rs1799794, 4541A/G, XRCC3. The MeSH term and entry terms of polymorphism were genetic polymorphism [MeSH terms]; polymorphisms, genetic; genetic polymorphisms; genetic polymorphism; polymorphism (genetics); polymorphisms (genetics); polymorphism, single nucleotide; nucleotide polymorphism, single; nucleotide polymorphisms, single; polymorphisms, single nucleotide; single nucleotide polymorphisms; polymorphisms; polymorphism; variant; mutation; single nucleotide polymorphism; SNP. The MeSH term and entry terms of cancer were neoplasm [MeSH terms], neoplasms, neoplasia, neoplasias, neoplasm, tumors, tumor, cancer, cancers, carcinoma, carcinogenesis, tumour. Furthermore, we refined the search results of related studies by looking at the list of references included in each article.

### Selection criteria

Relevant studies were included in accordance with the inclusion criteria and exclusion criteria, which were similar to those described in the previous study (PMID: 30867406). Original case–control study focused on the relationship between rs1799794 and cancer risk with the frequency of XRCC3 rs1799794 mutant genotypes were included. While conference abstracts or reports, reviews or meta-analyses, republished articles, and studies with insufficient data were excluded.

### Data extraction and quality assessment

The following data from each selected article were collected: the surname of the first author, the publication year, country, ethnicity, cancer types, and methods of genotyping XRCC3 rs1799794 polymorphism. The quality of eligible case–control studies was estimated using the Newcastle–Ottawa Scale [35].

### Statistical analysis

The relationship between XRCC3 rs1799794 polymorphisms and cancer risk were evaluated using odds ratios (ORs) and 95% confidence intervals (CI) under five genetic models (G vs. A, GG vs. AA, GG + GA vs. AA, GG vs. GA + AA, GA vs. AA).as previous study. If P < 0.05 or the 95% CI did not include 1, the result was considered statistically significant. Cochran’s Q with chi-square (with P_Q_) and the Higgins I^2^ test were used to determine heterogeneity in between-study variability. When P_Q_ < 0.1 or I^2^ > 25% indicated significant heterogeneity [36–38], we analyzed the data using a random effects model [39]. If the opposite held, a fixed effects model was chosen. We also performed subgroup analyses and a sensitivity analysis to explore sources of heterogeneity. Subgroup analyses stratified studies by cancer type (ovarian cancer, acute lymphoblastic leukemia, breast cancer, thyroid cancer, bladder cancer, lung cancer, other), ethnicity (Arabian, Asian, Caucasian, mixed), sample size (< 100, > 100), the publication year (≤ 2010, > 2010), detection method (PCR–RFLP, sequencing, TaqMan, PCR, ND, other), and source of control (HB, PB, mixed, nested). We assessed publication bias using funnel plots and Egger’s test (P < 0.05). Statistical calculations were performed with Stata 14.0.

## Results

### Literature search and study characteristics

**Fig. 1:**
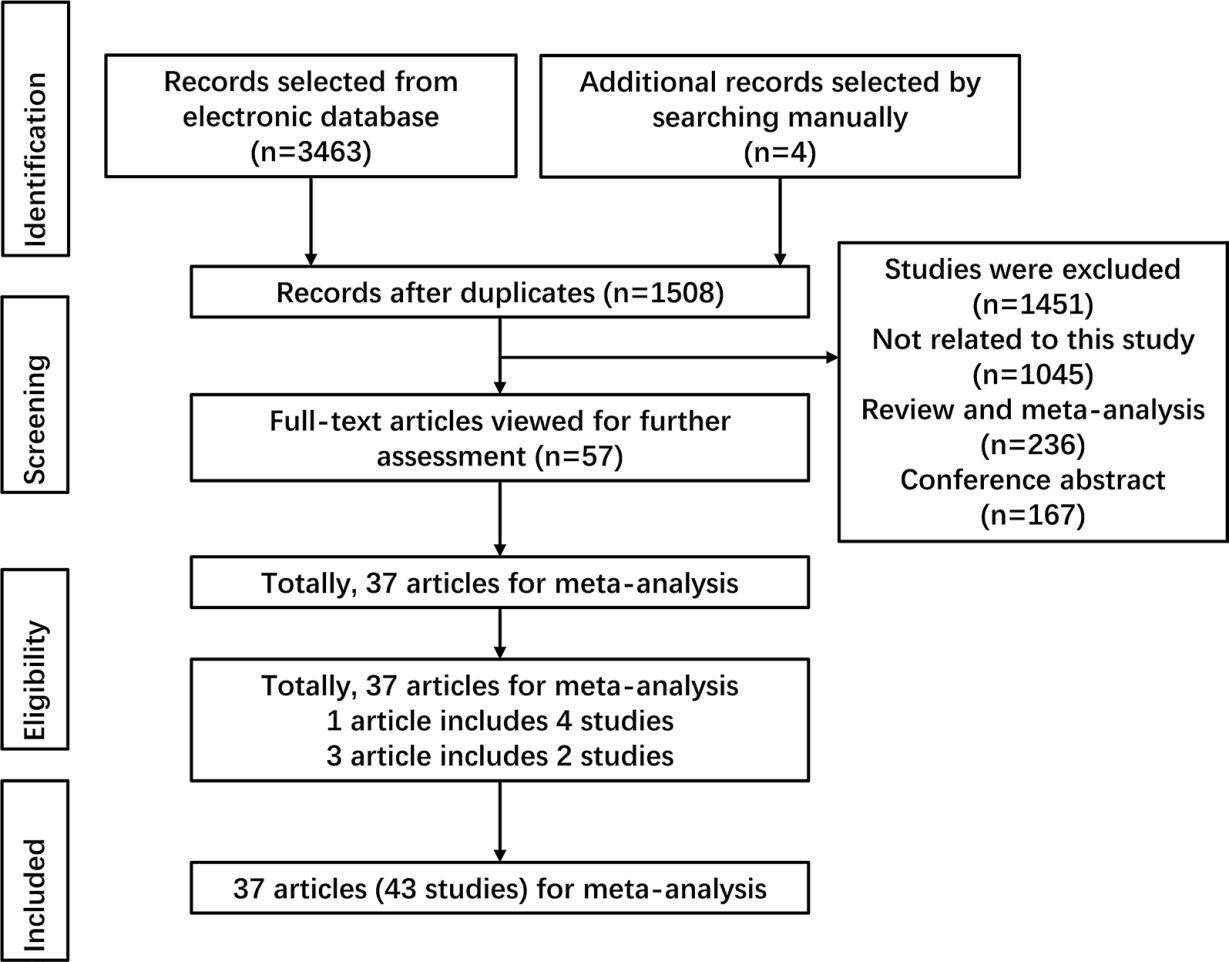
Flow chart of study selection

Finally, 3,467 potentially relevant published works were identified (997 in PubMed, 27 in the Cochrane library, 855 in Embase, 696 in Scopus, and 889 in Web of Science). Of these, duplicates (1959) and works not related to cancer and rs1799794 polymorphism (1451) were excluded. Then 23 of these studies were excluded after reviewing full texts. The remaining 37 works (43 studies) were included in this meta-analysis [10, 40–75]. Because two studies in Auranen et al. [10] were duplicated in Quaye et al. [62], we only extracted data from these studies from Auranen et al. [10] to avoid duplication; thus, one article included four studies [66], and three articles included two studies each [10, 68, 70]. The flow chart of the literature selection process is shown in Fig. 1.

There were a total of 23,537 cases and 30,649 controls in these 37 works, and 3 were conducted among Arabians [40, 48, 55], 14 among Asians [41, 42, 45–47, 49, 50, 53, 54, 56, 58, 59, 66, 67], and 24 among Caucasians [10, 43, 44, 51, 52, 57, 60–62, 64, 66, 69–75]; 2 were conducted among mixed populations [63, 65]. In addition, in terms of cancer type, ovarian cancer (n = 4) [10, 40, 62], acute lymphoblastic leukemia (n = 3) [41, 52, 57], breast cancer (n = 13) [44, 48, 49, 55, 61, 66, 68, 72, 74], thyroid cancer (n = 4) [42, 46, 47, 67], bladder cancer (n = 4) [45, 63, 65, 69], lung cancer (n = 3) [53, 59, 71], and other cancer (hepatocellular cancer, leiomyoma, nasopharyngeal carcinoma, osteosarcoma, oral cancer, glioma, head and neck cancer, myeloma, endometrial cancer, colorectal adenoma, melanoma skin cancer) [43, 50, 51, 54, 56, 58, 60, 64, 70, 73, 75] were studied. The basic information of each study is presented in Table 1. And we took sensitivity analysis for studies that do not conform to HWE.

**Table 1:** Characteristics of the individual studies included in the meta-analysis

Author	Year	Country	Ethnicity	Cancer type	Genotyping method	Control	Cases/control	Cases			Control			HWE	NOS
								aa	ag	gg	aa	ag	gg		
Mackawy	2019	Egypt	Arabian	Ovarian carcinoma	PCR–RFLP	HB	50/20	14	20	16	4	6	10	0.128	6
Pei	2018	China	Asian	Acute lymphoblastic leukemia	PCR–RFLP	PB	266/266	55	144	67	53	150	63	0.035	7
Al Zoubi	2017	Italy	Caucasian	Breast cancer	Sequencing	HB	23/16	8	14	1	11	5	0	0.459	7
De Mattia	2017	Italy	Caucasian	Hepatocellular cancer	TaqMan	HB	192/192	128	52	12	137	49	5	0.806	7
Sarwar	2017	Pakistan	Asian	Thyroid cancer	ARMS-PCR	HB	456/400	289	90	77	297	65	38	< 0.001	7
Yan	2016	China	Asian	Yhyroid carcinoma	PCR	HB	276/403	116	127	33	202	161	40	0.345	8
Zhu	2016	China	Asian	Bladder cancer	TaqMan	HB	184/260	72	53	59	69	142	49	0.111	7
Ali	2016	Saudi Arabia	Arabian	Breast cancer	PCR–RFLP	HB	143/145	102	40	1	93	28	24	< 0.001	7
Chang	2015	China	Asian	Leiomyoma	PCR–RFLP	HB	166/474	35	91	40	93	268	113	0.004	7
Chen	2015	China	Asian	Lung cancer	PCR–RFLP	HB	358/716	70	202	86	147	395	177	0.007	7
Liu	2015	China	Asian	Nasopharyngeal carcinoma	PCR–RFLP	HB	176/880	33	99	44	179	489	212	0.001	7
Su	2015	China	Asian	Breast cancer	PCR–RFLP	HB	1232/1232	239	696	297	254	668	310	0.002	8
Al Zoubi	2015	Jordan	Arabian	Breast cancer	Sequencing	HB	46/31	16	28	2	21	9	1	0.976	7
Yuan	2015	China	Asian	Papillary thyroid cancer	PCR	HB	183/367	77	84	22	184	147	36	0.406	6
Goričar	2015	Slovenia	Caucasian	Acute lymphoblastic leukemia	TaqMan	PB	121/184	89			117			≥ 0.050	8
Goričar	2015	Slovenia	Caucasian	Osteosarcoma	TaqMan	PB	79/373	47			247			ND	7
Smolkova	2014	Germany	Caucasian	Acute lymphoblastic leukaemia	TaqMan	HB	460/547	286	155	19	340	183	24	0.921	7
TSAI	2014	China	Asian	Oral cancer	PCR–RFLP	HB	788/956	155	438	195	195	532	229	< 0.001	7
He	2013	China	Asian	Lung cancer	PCR	HB	507/661	180	230	97	184	313	164	0.181	7
Zhao	2013	China	Asian	Glioma	TaqMan	HB	384/384	83	201	100	95	181	108	0.271	7
Gresner	2012	Poland	Caucasian	Head and neck cancer	PCR–RFLP	PB	81/100	45	31	5	59	34	7	0.497	8
VRAL	2011	Belgium	Caucasian	Breast cancer	PCR–RFLP or SnapShot technique	HB	343/172	220	108	15	117	52	3	0.304	5
Quaye	2009	mixed	Caucasian	Ovarian cancer	TaqMan	PB	1461/2307	940	484	37	1505	713	89	0.691	8
Andrew	2009	USA	Mixed	Bladder cancer	PCR–RFLP	PB	342/559	190			333			ND	7
Hayden	2007	Germany, Italy, Spain, Ireland,France, Czech Republic and the Irish	Caucasian	Myeloma	TaqMan	Mixed	302/257	189	100	13	153	91	13	0.911	8
Ni	2006	China	Asian	Thyroid carcinoma	PCR–RFLP	HB	191/201	66	81	44	62	94	45	0.411	7
Garcıa-Closas	2006	Poland	Caucasian	Breast cancer	ND	PB	1920/2218	1210	632	78	1386	736	96	0.891	8
Garcıa-Closas	2006	USA	Caucasian	Breast cancer	ND	PB	1564/1264	980	521	63	837	357	52	0.079	8
Wu	2006	USA	Mixed	Bladder cancer	PCR–RFLP	HB	599/595	402	185	12	398	185	12	0.072	8
Paul Pharoah ICRC-Thai	2006	Thailand	Asian	Breast cancer	ND	PB	465/389	153	217	95	135	182	72	0.441	8
Paul Pharoah SEARCH	2006	UK	Caucasian	Breast cancer	ND	PB	2790/3642	1808	889	93	2388	1113	141	0.427	8
Paul Pharoah Sheffield	2006	UK	Caucasian	Breast cancer	ND	HB	1185/1159	781	369	35	755	353	51	0.238	8
Paul Pharoah USRTS	2006	USA	Caucasian	Breast cancer	ND	Nested	718/1049	458	224	36	650	356	43	0.509	8
Auranen1	2005	US FROC	Caucasian	Ovarian cancer	TaqMan	PB	325/417	204	112	9	267	133	17	0.932	8
Auranen2	2005	UK RMH/YOV	Caucasian	Ovarian cancer	TaqMan	PB	301/1808	194	95	12	1196	535	77	0.083	8
Matullo	2005	Italy	Caucasian	Bladder cancer	PCR–RFLP	HB	316/315	207	98	11	201	102	12	0.833	7
Han	2004	USA	Caucasian	Breast cancer	TaqMan	PB	991/1291	630	322	39	865	372	54	0.084	8
Han	2004	USA	Caucasian	Endometrial cancer	TaqMan	PB	220/663	140	73	7	438	200	25	0.716	8
Jacobsen	2004	Denmark	Caucasian	Lung cancer	PCR	Nested	256/269	111	116	29	108	127	34	0.724	8
Tranah	2004	USA (NHS)	Caucasian	Colorectal adenoma	TaqMan	Nested	556/557	256	212	58	250	222	54	0.65	8
Tranah	2004	USA (HPFS)	Caucasian	Colorectal adenoma	TaqMan	Nested	376/725	180	155	31	329	303	73	0.793	8
Kuschel	2002	UK	Caucasian	Breast cancer	TaqMan	PB	1828/1808	1176	581	71	1196	535	77	0.083	8
Winsey	2000	UK	Caucasian	Melanoma skin cancer	PCR-SSP	HB	125/211	5	47	73	8	80	122	0.245	7

### Meta-analysis and subgroup analyses

The value of I^2^ in the five genetic models was greater than 25%, and P_Q_ < 0.10, so pooled ORs for the five genetic models were calculated with a random effects model. There was no obvious correlation between rs1799794 and cancer risk (P_Z_ > 0.05; Table 2).

**Table 2:** The results of the meta-analyses under different genetic models for all studies

Genetic model	Number	I^2^ (%)	P_H_	OR (95% CI)	P_Z_
G VS A	40	47.50	0.001	1.02(0.98–1.07)	0.377
GG VS AA	40	30.20	0.039	0.98(0.89–1.08)	0.713
GG + GA VS AA	43	40.0	0.004	1.04(0.98–1.09)	0.207
GG VS GA + AA	40	34.10	0.02	0.98(0.90–1.07)	0.696
GA VS AA	40	39.40	0.006	1.04(0.99–1.11)	0.134

Subgroup analyses were then performed based on cancer type, ethnicity, detection method, the publication year, source of control, and sample size to investigate sources of heterogeneity (Table 3). In the subgroup analysis based on cancer type, a significantly increased risk for thyroid cancer was observed in the five models (G vs. A: OR = 1.27, 95% CI = 1.01–1.61, I^2^ = 71.2%; GG + AG vs. AA: OR = 1.36, 95% CI = 1.15–1.61, I^2^ = 55.4%; GG vs. AA + AG: OR = 1.38, 95% CI = 1.09–1.75, I^2^ = 29.8%; GG vs. AA: OR = 1.50, 95% CI = 1.17–1.93, I^2^ = 45.7%; AG vs. AA: OR = 1.27, 95% CI = 1.05–1.53, I^2^ = 33.2%), a significantly increased risk for breast cancer was found in the heterozygous model (OR = 1.08, 95% CI = 1.02–1.13, I^2^ = 42.3%), and a decreased risk for ovarian cancer was found in the recessive model and homozygous model (GG vs. AA + AG: OR = 0.69, 95% CI = 0.51–0.93, I^2^ = 0.0%; GG vs. AA: OR = 0.71, 95% CI = 0.53–0.96, I^2^ = 0.0%).

**Table 3:** Results of meta-analysis for polymorphisms in different subgroups and cancer susceptibility

Comparison	Subgroup	Number	I^2^	P_H_	P_Z_	OR (95% CI)
G VS A	Ethnicity					
	Arabian	3	84.9%	0.001	0.752	0.86 (0.33–2.23)
	Asian	14	64.8%	P < 0.001	0.255	1.05 (0.96–1.15)
	Caucasian	22	0.0%	0.661	0.502	1.01 (0.98–1.05)
	Mixed	1	NA	NA	0.940	0.99 (0.80–1.23)
	Cancer type					
	Ovarian cancer	4	0.0%	0.547	0.848	0.99 (0.90–1.09)
	Acute lymphoblastic leukemia	2	0.0%	0.887	0.979	1.00 (0.85–1.18)
	Breast cancer	13	58.6%	0.004	0.494	1.03 (0.95–1.10)
	Thyroid cancer	4	71.2%	0.015	0.043	1.27 (1.01–1.61)
	Bladder cancer	3	0.0%	0.921	0.815	0.98 (0.85–1.13)
	lung cancer	3	60.1%	0.082	0.166	0.88 (0.74–1.05)
	Others	11	0.0%	0.902	0.822	1.01 (0.91–1.08)
	Method					
	PCR–RFLP	12	22.3%	0.225	0.657	0.99 (0.93–1.05)
	Sequencing	2	0.0%	0.828	0.004	2.60 (1.37–4.94)
	TaqMan	13	0.0%	0.886	0.475	1.02 (0.97–1.07)
	PCR	4	82.4%	0.001	0.913	1.02 (0.78–1.33)
	ND	6	14.6%	0.321	0.663	1.01 (0.96–1.06)
	Others	3	68.3%	0.043	0.089	1.32 (0.96–1.82)
	Source of control					
	HB	23	66.0%	P < 0.001	0.445	1.03 (0.95–1.13)
	PB	12	0.0%	0.892	0.135	1.03 (0.99–1.08)
	Mixed	1	NA	NA	0.442	0.89 (0.67–1.19)
	Nested	4	0.0%	0.874	0.294	0.95 (0.86–1.05)
	Sample size					
	< 100	3	77.1%	0.013	0.419	1.54 (0.54–4.43)
	> 100	37	43.7%	0.003	0.424	1.02 (0.98–1.07)
	Year					
	≤ 2010	20	0.0%	0.910	0.700	1.01 (0.97–1.04)
	> 2010	20	69.5%	0.000	0.272	1.06 (0.96–1.17)
GG + AG VS AA	Ethnicity					
	Arabian	3	79.8%	0.007	0.739	1.21 (0.39–3.76)
	Asian	14	64.4%	P < 0.001	0.547	1.04 (0.91–1.20)
	Caucasian	24	0.6%	0.453	0.119	1.03 (0.99–1.08)
	Mixed	2	0.0%	0.620	0.765	1.03 (0.85–1.24)
	Cancer type					
	Ovarian cancer	4	0.0%	0.887	0.439	1.05 (0.93–1.17)
	Acute lymphoblastic leukemia	3	24.4%	0.267	0.397	0.90 (0.75–1.12)
	Breast cancer	13	47.0%	0.031	0.037	1.06 (0.98–1.15)
	Thyroid cancer	4	55.4%	0.081	0.033	1.36 (1.15–1.61)
	Bladder cancer	4	59.1%	0.062	0.370	0.89 (0.70–1.14)
	Lung cancer	3	51.2%	0.129	0.207	0.85 (0.66–1.09)
	Others	12	0.0%	0.910	0.597	1.03 (0.93–1.13)
	Method					
	PCR–RFLP	13	0.0%	0.965	0.840	1.01 (0.92–1.11)
	Sequencing	2	0.0%	0.956	0.001	4.00 (1.82–8.80)
	TaqMan	15	29.2%	0.137	0.269	1.04 (0.97–1.10)
	PCR	4	81.0%	0.001	0.862	1.03 (0.72–1.48)
	ND	6	28.0%	0.225	0.360	1.03 (0.97–1.09)
	Others	3	16.0%	0.304	0.051	1.45 (1.15–1.82)
	Source of control					
	HB	23	58.4%	P < 0.001	0.397	1.05 (0.94–1.18)
	PB	15	0.0%	0.656	0.015	1.06 (1.01–1.12)
	Mixed	1	NA	NA	0.461	0.88 (0.63–1.24)
	Nested	4	0.0%	0.979	0.190	0.92 (0.82–1.04)
	Sample size					
	< 100	3	65.8%	0.054	0.179	2.23 (0.69–7.21)
	> 100	40	32.9%	0.025	0.234	1.03 (0.98–1.09)
	Year					
	≤ 2010	21	0.0%	0.815	0.166	1.03 (0.99–1.08)
	> 2010	22	62.0%	0.000	0.322	1.07 (0.94–1.22)
GG VS AA + AG	Ethnicity					
	Arabian	3	73.9%	0.022	0.218	0.28 (0.04–2.13)
	Asian	14	52.7%	0.011	0.253	1.08 (0.95–1.23)
	Caucasian	22	0.0%	0.806	0.056	0.91 (0.82–1.00)
	Mixed	1	NA	NA	0.987	0.99 (0.44–2.23)
	Cancer type					
	Ovarian cancer	4	0.0%	0.678	0.014	0.69 (0.51–0.93)
	Acute lymphoblastic leukemia	2	0.0%	0.698	0.818	1.04 (0.75–1.45)
	Breast cancer	13	35.7%	0.097	0.101	0.92 (0.83–1.02)
	Thyroid cancer	4	29.8%	0.234	0.007	1.38 (1.09–1.75)
	Bladder cancer	3	52.3%	0.123	0.303	1.35 (0.76–2.37)
	Lung cancer	3	5.5%	0.347	0.062	0.83 (0.69–1.01)
	Others	11	0.0%	0.893	0.993	1.00 (0.88–1.13)
	Method					
	PCR–RFLP	12	18.3%	0.265	0.421	0.96 (0.86–1.06)
	Sequencing	2	0.0%	0.818	0.621	1.63 (0.23–11.46)
	TaqMan	13	41.3%	0.059	0.462	0.95 (0.84–1.08)
	PCR	4	44.2%	0.146	0.211	0.88 (0.71–1.08)
	ND	6	8.8%	0.360	0.363	0.94 (0.81–1.08)
	Others	3	60.9%	0.078	0.121	1.54 (0.89–2.64)
	Source of control					
	HB	23	55.0%	0.010	0.614	1.04 (0.90–1.20)
	PB	12	0.0%	0.862	0.111	0.91 (0.81–1.02)
	Mixed	1	NA	NA	0.674	0.84 (0.38–1.02)
	Nested	4	0.0%	0.536	0.967	1.00 (0.80–1.24)
	Sample size					
	< 100	3	0.0%	0.537	0.339	0.64 (0.26–1.59)
	> 100	37	36.9%	0.014	0.766	0.99 (0.90–1.07)
	Year					
	≤ 2010	20	0.0%	0.928	0.068	0.94 (0.83–1.01)
	> 2010	20	58.0%	0.001	0.374	1.08 (0.92–1.27)
GG VS AA	Ethnicity					
	Arabian	3	75.4%	0.017	0.338	0.33 (0.04–3.15)
	Asian	14	47.8%	0.024	0.279	1.08 (0.93–1.26)
	Caucasian	22	0.0%	0.812	0.083	0.91 (0.82–1.01)
	Mixed	1	NA	NA	0.981	0.99 (0.44–2.23)
	Cancer type					
	Ovarian cancer	4	0.0%	0.705	0.028	0.71 (0.53–0.96)
	Acute lymphoblastic leukemia	2	0.0%	0.836	0.961	0.99 (0.67–1.47)
	Breast cancer	13	37.7%	0.082	0.311	0.94 (0.85–1.05)
	Thyroid cancer	4	45.7%	0.137	0.001	1.50 (1.17–1.93)
	Bladder cancer	3	0.0%	0.860	0.773	1.06 (0.72–1.55)
	Lung cancer	3	53.1%	0.119	0.019	0.79 (0.56–1.11)
	Others	11	0.0%	0.884	0.798	1.02 (0.88–1.19)
	Method					
	PCR–RFLP	12	10.7%	0.340	0.591	0.96 (0.85–1.10)
	Sequencing	2	0.0%	0.837	0.264	3.09 (0.43–22.45)
	TaqMan	13	0.0%	0.701	0.297	0.93 (0.81–1.07)
	PCR	4	73.8%	0.010	0.937	0.98 (0.61–1.58)
	ND	6	2.7%	0.399	0.436	0.94 (0.82–1.09)
	Others	3	0.0%	0.409	P < 0.001	1.97 (1.36–2.87)
	Source of control					
	HB	23	52.8%	0.002	0.628	1.04 (0.88–1.24)
	PB	12	0.0%	0.911	0.185	0.92 (0.82–1.04)
	Mixed	1	NA	NA	0.604	0.81 (0.36–1.80)
	Nested	4	0.0%	0.553	0.737	0.96 (0.76–1.21)
	Sample size					
	< 100	3	18.0%	0.295	0.796	0.87 (0.31–2.48)
	> 100	37	32.5	0.031	0.733	0.98 (0.89–1.08)
	Year					
	≤ 2010	20	0.0%	0.961	0.070	0.91 (0.82–1.01)
	> 2010	20	55.2%	0.002	0.356	1.06 (0.96–1.17)
AG VS AA	Ethnicity					
	Arabian	3	54.9%	0.109	0.174	1.76 (0.78–3.95)
	Asian	14	65.7%	P < 0.001	0.906	1.01 (0.86–1.18)
	Caucasian	22	0.0%	0.631	0.023	1.05 (1.01–1.10)
	Mixed	1	NA	NA	0.937	0.99 (0.77–1.27)
	Cancer type					
	Ovarian cancer	4	0.0%	0.998	0.145	1.09 (0.97–1.22)
	Acute lymphoblastic leukemia	2	0.0%	0.747	0.893	0.98 (0.78–1.24)
	Breast cancer	13	42.3%	0.054	0.006	1.08 (1.02–1.13)
	Thyroid cancer	4	33.2%	0.213	0.012	1.27 (1.05–1.53)
	Bladder cancer	3	87.1%	P < 0.001	0.038	0.71 (0.41–1.23)
	Lung cancer	3	26.7%	0.255	0.132	0.87 (0.73–1.04)
	Others	11	0.0%	0.935	0.710	1.02 (0.92–1.13)
	Method					
	PCR–RFLP	12	0.0%	0.981	0.590	1.03 (0.93–1.14)
	Sequencing	2	0.0%	0.946	0.001	4.00 (1.79–8.94)
	TaqMan	13	57.1%	0.006	0.696	1.02 (0.92–1.14)
	PCR	4	72.9%	0.011	0.780	1.05 (0.76–1.44)
	ND	6	35.1%	0.173	0.205	1.04 (0.98–1.11)
	Others	3	0.0%	0.577	0.089	1.25 (0.97–1.63)
	Source of control					
	HB	23	56.0%	0.001	0.421	1.05 (0.93–1.18)
	pb	12	0.0%	0.803	0.002	1.09 (1.03–1.15)
	MIXED	1	NA	NA	0.518	0.89 (0.62–1.27)
	Nested	4	0.0%	0.989	0.160	0.91 (0.80–1.04)
	Sample size					
	< 100	3	31.6%	0.232	0.003	2.82 (1.42–5.57)
	> 100	37	32.9%	0.029	0.153	1.04 (0.99–1.10)
	Year					
	≤ 2010	20	0.0%	0.667	0.047	1.05 (1.00–1.10)
	> 2010	20	60.8%	0.000	0.278	1.08 (0.94–1.25)

In the subgroup analysis based on ethnicity, rs1799794 was associated with increased cancer risk in the Caucasian population according to the heterozygous model (AG vs. AA: OR = 1.05, 95% CI = 1.01–1.10, I^2^ = 0.0%). In the subgroup analysis based on source of control, we found a significantly increased risk for PB (population based) in the dominant model and heterozygous model (GG + AG vs. AA: OR = 1.06, 95% CI = 1.01–1.12, I^2^ = 0.0%; AG vs. AA: OR = 1.09, 95% CI = 1.03–1.15, I^2^ = 0.0%). In the subgroup analysis based on detection method, sequencing was associated with a significantly increased cancer risk in the allele model, dominant model, and heterozygous model (G vs. A: OR = 2.60, 95% CI = 1.37–4.94, I^2^ = 0.0%; GG + AG vs. AA: OR = 4.00, 95% CI = 1.82–8.80, I^2^ = 0.0%; AG vs. AA: OR = 4.00, 95% CI = 1.79–8.94, I^2^ = 0.0%). In the subgroup analysis based on sample size, AG carriers were 2.82 times more likely to develop cancer than AA carriers (95% CI = 1.42–5.57, P_Z_ = 0.003). In the subgroup analysis based on the publication year, studies published before 2010 showed that AG carriers were 1.05 times more likely to develop cancer than AA carriers (95% CI = 1.00–1.10, P_Z_ = 0.047).

### Publication bias

**Fig. 2:**
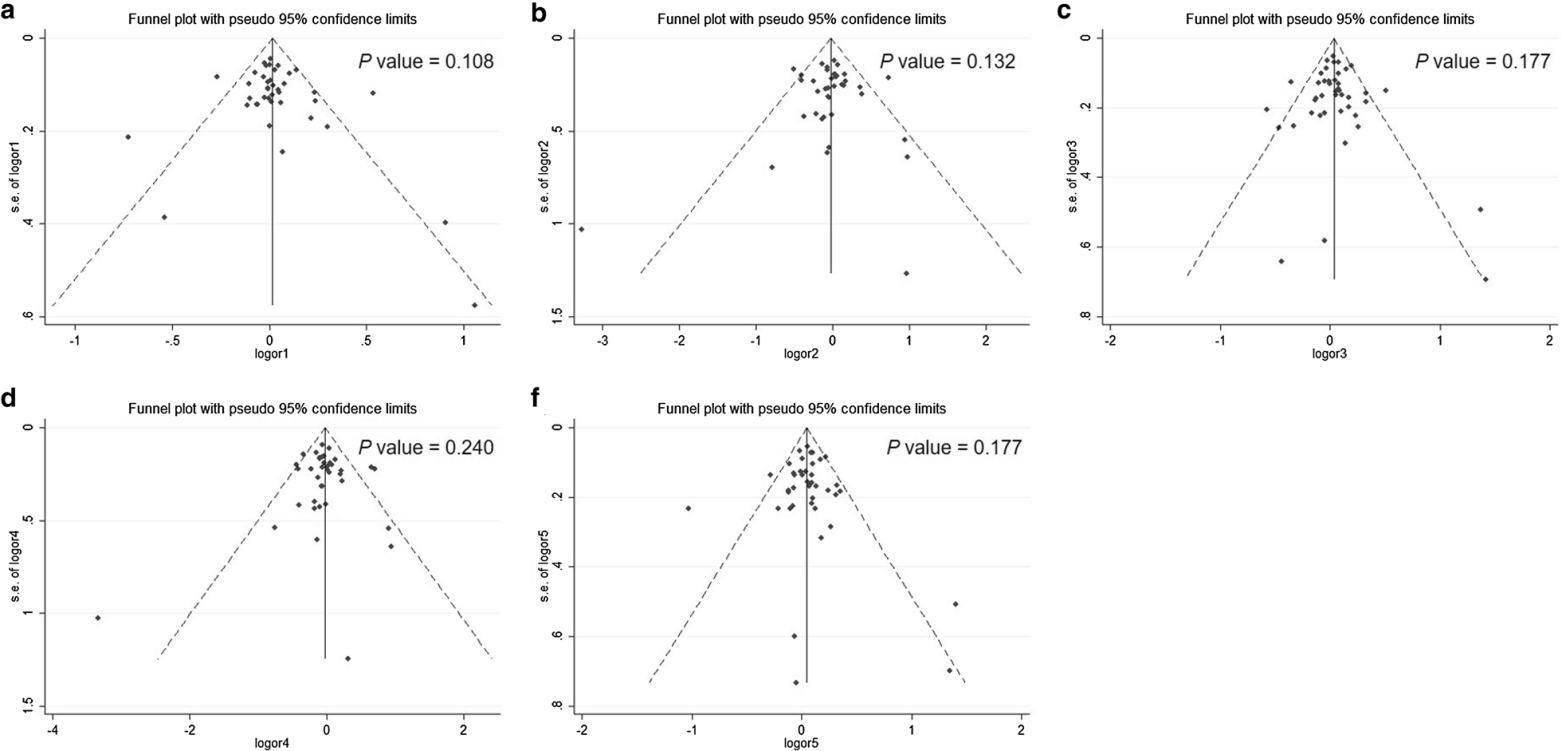
Funnel plots for the test of publication bias for the five genetic models

The shape of the funnel plots (Fig. 2) and Egger’s test (allele: P = 0.108, dominant: P = 0.177, recessive: P = 0.240, homozygous: P = 0.132, heterozygous: P = 0.177) showed no publication bias.

### Sensitivity analysis

Eight studies [41, 42, 48–50, 53, 54, 56] had P_HWE_ < 0.05, but for two studies [51, 63] P_HWE_ was not available. We compared the combined results before and after excluding these 10 studies and there were slight changes in the results. When the subgroup analysis was performed according to cancer type, there were no significant associations between rs1799794 polymorphism and increased risk for thyroid cancer in the recessive model, homozygous model, or heterozygous model (GG vs. AA + AG: OR = 1.16, 95% CI = 0.87–1.55, I^2^ = 0.0%; GG vs. AA: OR = 1.24, 95% CI = 0.90–1.69, I^2^ = 0.0%; AG vs. AA: OR = 1.22, 95% CI = 0.98–1.51, I^2^ = 49.4%), and rs3116496 was related to a decreased risk for lung cancer in the five models (A vs. G: OR = 0.80, 95% CI = 0.70–0.92, I^2^ = 18.1%; GG + AG vs. AA: OR = 0.76, 95% CI = 0.62–0.93, I^2^ = 4.9%; GG vs. AA + AG: OR = 0.75, 95% CI = 0.59–0.96, I^2^ = 0.0%; GG vs. AA: OR = 0.65, 95% CI = 0.49–0.87, I^2^ = 0.0%; AG vs. AA: OR = 0.80, 95% CI = 0.64–0.99, I^2^ = 0.0%); no changes were observed for the other cancers. No significant changes were found in the subgroup analyses by ethnicity and source of control.

## Discussion

Our study shows that XRCC3 rs1799794 is irrelevant to cancer risk. In addition, the risk for thyroid cancer and breast cancer increase significantly in patients with rs1799794, and Caucasian populations are more likely to develop these cancers while having a decreased risk for ovarian cancer. We excluded articles that did not conform to HWE and reanalyzed the data. Compared to the previous results, rs3116496 was related to a decreased risk for lung cancer in the five models, although the other results were not much changed (data not shown).

Moderate heterogeneity was found in this meta-analysis. First, we used random models when significant heterogeneity. Second, we performed subgroup analyses to explore sources of heterogeneity. As shown in Table 3, in the subgroup analysis based on ethnicity, heterogeneity increased in Arabian/Asian populations but was 0% in Caucasian populations, which suggests that ethnicity may be a factor in heterogeneity. Furthermore, we analyzed studies stratified by cancer type, detection method, source of control, and sample size. Ethnicity, cancer type, source of control, and sample size may be the source of inter-research heterogeneity. In addition, a sensitivity analysis suggested that the current findings were reliable.

To date, five meta-analyses of the impact of rs1799794 on cancer risk have been performed [28, 30, 31, 33, 34] on rs1799794 and susceptibility to pan-cancer [28], breast cancer [30, 34], bladder cancer [33], and ovarian cancer [31]. To the best of our knowledge, ours is currently the most comprehensive meta-analysis of correlations between rs1799794 polymorphisms and cancer. There are many differences between the results of this study and previous studies. According to Qiu et al.’s research on rs1799794 and susceptibility to breast cancer, which included four studies in three papers, rs1799794 was associated with a statistically significant increase in cancer risk in the dominant model (GG + AG vs. AA: OR = 1.09, 95% CI = 1.01–1.17, P_H_ = 0.15), whereas our results showed an increased risk for breast cancer in AG carriers, different from the protective effect found previously [48]. In addition, our study found that the G allele might be a dominant gene and found an increased risk for thyroid cancer.

Our study included a large number of samples and conducted a stratified analysis, which played an important role in the reliability of the research results. At the same time, there are problems that cannot be ignored: the presence of heterogeneity that may due to ethnicity, source of control, status, or cancer type; the lack of relevant data published in other languages and evaluation of the interaction between cancer-related factors.

## Conclusion

In conclusion, this meta-analysis found no association between XRCC3 rs1799794 and cancer risk, but XRCC3 rs1799794 was associated with breast cancer and thyroid cancer as well as with Caucasian populations. In addition, detection method, source of control, and sample size played a role in heterogeneity and in the results. Well-designed large-scale studies are required to further evaluate the results.

## Data Availability

All data generated or analyzed during this study are included in this manuscript.

## Abbreviations

SNP: Single nucleotide polymorphism
XRCC3: X-ray repair cross-complementing group 3
HR: Homologous recombination
OR: Odds ratio
CI: Confidence interval
